# Cardiovascular Autonomic Neuropathy and its Association with Cardiovascular and All-cause Mortality in Patients with End-stage Renal Disease

**DOI:** 10.7759/cureus.3243

**Published:** 2018-08-31

**Authors:** Syed Rizwan A Bokhari, Faisal Inayat, Ali Jawa, Hafeez Ul Hasan Virk, Muhammad Awais, Nadeem Hussain, Ghias Ul Hassan, Hafiz Ijaz Ahmad, Hammad S Chaudhry, Abdullah Adil, Ali Haider, Vincent M Figueredo, Janani Rangaswami, Muhammad Zaman Khan Assir

**Affiliations:** 1 Department of Nephrology and Hypertension, Tulane University School of Medicine, New Orleans, USA; 2 Internal Medicine, Allama Iqbal Medical College, Lahore, PAK; 3 Department of Medicine, Shaheed Zulfiqar Ali Bhutto Medical University, Islamabad, PAK; 4 Department of Cardiovascular Diseases, Einstein Heart and Vascular Institute, New York, USA; 5 Department of Medicine, Prince Sultan Military Medical City, Riyadh, SAU; 6 Department of Medicine, Allama Iqbal Medical College, Lahore, PAK; 7 Department of Medicine, Ameer Ud Din Medical College, Lahore, PAK; 8 Department of Nephrology, Allama Iqbal Medical College, Lahore, PAK; 9 Department of Medicine, Allama Iqbal Medical College/Jinnah Hospital, Lahore, PAK; 10 Depertment of Medicine, Allama Iqbal Medical College, Lahore, PAK; 11 Department of Medicine, Allama Iqbal Medical College, Jhang, PAK; 12 Department of Cardiovascular Diseases, Einstein Medical Center, Philadelphia, USA; 13 Division of Nephrology, Einstein Medical Center, Philadelphia, USA; 14 Internal Medicine, Allama Iqbal Medical College/Jinnah Hospital, Lahore, PAK

**Keywords:** cardiovascular autonomic neuropathy, cardiovascular and all-cause mortality, end-stage renal disease, risk assessment

## Abstract

Background

End-stage renal disease frequently leads to increased cardiovascular mortality. Cardiovascular autonomic neuropathy (CAN) may be predictive of cardiac arrhythmias and sudden cardiac death in patients with end-stage renal disease.

Methods

A total of 70 patients with end-stage renal disease were included in the study. The assessment of cardiac dysautonomia was based on the four standardized tests performed at the baseline and, again, at the end of the study. The criteria for CAN included at least two abnormal test results.

Results

Fifty of 70 patients completed the study and were followed-up after one year. Out of the 50 patients, 44 (88%) had CAN at baseline. Twelve (24%) patients died at the one-year follow-up. Sudden cardiac death was reported in seven out of 12 (58%) patients. All seven patients who died had high dysautonomia scores (three abnormal tests) at the baseline. There was a significantly higher percentage of patients with all four abnormal tests amongst patients who died of any cause (56% vs. 17%; RR 6.07, 95% CI 1.29-28.49; p-value 0.02) or due to sudden cardiac death (43% vs. 10.5%; RR 6.37, 95% CI 1.03-39.36; p-value 0.04). All five patients who did not have CAN at the baseline developed this abnormality on repeat testing after one year.

Conclusion

The prevalence of CAN in patients with end-stage renal disease on maintenance hemodialysis was significantly higher. CAN was an independent predictor of all-cause and cardiovascular mortality, which highlights it as a risk stratification tool in patients with end-stage renal disease.

## Introduction

Chronic kidney disease (CKD) is associated with high morbidity and mortality throughout the continuum from early disease to advanced stages requiring dialysis. The approximate incidence of this illness ranges from 13% to 16% in Europe and the United States, respectively [1]. Patients with CKD demonstrate a predilection to die prematurely, which has largely been attributed to the death from cardiovascular (CV) disease. Recently, increased CV and all-cause mortality in patients with CKD have become an interesting topic that merits investigation [1].

Left ventricular hypertrophy, systolic dysfunction, accelerated vascular and valvular calcification and extremes of systolic blood pressure have been documented as independent risk factors for CV events in patients with end-stage renal disease (ESRD) [2]. The cardiac autonomic function is an important parameter that deranges in CKD. The presence of cardiac autonomic neuropathy (CAN) has been reported independently of the presence of diabetes in patients on hemodialysis [3,4]. The clinical manifestations of CAN in subjects with kidney disease include hypotension during dialysis, which is attributed to impaired ability to maintain the systemic blood pressure following ultrafiltration as well as impaired homeostatic responses. In such chronically hypotensive individuals, a significant down-regulation of alpha- and beta-adrenergic receptors occur. Furthermore, CAN may also culminate in resting tachycardia, exercise intolerance, orthostatic hypotension, silent ischemia, silent myocardial infarction, and increased incidence of arrhythmic complications leading to a higher incidence of sudden cardiac death (SCD) [5].

Predicting the outcomes of patients with CKD carries paramount importance not only for the long-term management of renal disorders but also for effective channelization of healthcare delivery efforts. Given the phenotype of CV disease in patients with advanced CKD, several markers of prognosis are studied. High coronary calcification burden, increased aortic stiffness, anemia, hypoalbuminemia, oxidative stress, electrolyte imbalance, and low fetuin-A levels have previously been linked to increased mortality in patients with ESRD [6-8]. However, recent studies implicated a limited benefit for these biomarkers [8]. Therefore, improvement in the risk assessment by devising additional efficient prognostic tools is clearly warranted in patients with ESRD. The aim of our study was to prospectively evaluate the prevalence of CAN and its association with CV and all-cause mortality in patients with ESRD on maintenance hemodialysis. The preliminary form of the data was presented as an abstract (Abstract: Ahmad HI, Asif A, Bokhari SRA, Assir MZK, Awais M, Nasir S. ''Cardiac Dysautonomia in Patients with End-Stage Renal Disease on Hemodialysis'', American Society of Nephrology Kidney Week, November 08-13, 2011 in Philadelphia, Pennsylvania). 

## Materials and methods

Patients

A total of 70 patients with ESRD were included in the study. Patients were recruited consecutively from the Outpatient Hemodialysis Center, Department of Nephrology and Hypertension, Jinnah Hospital, Lahore, Pakistan. Established questionnaires were used for the data collection regarding patient demographics and medical history, vital sign examination findings, the results of cardiac dysautonomia testing, and mortality information of the participants at the end of one year. After the initial assessment, 70 participants aged 16 years and above of either gender, undergoing regular hemodialysis for at least six months, with or without diabetes, were enrolled in the study. Demographic and clinical data were collected at the bedside, during their hospital visit for hemodialysis. The patients were tested for CAN at the baseline. At the follow-up after one year, the tests were repeated to ascertain any change from the baseline. The number of SCDs among the cohort during the observation period was also recorded and correlated with the presence of CAN.

Assessment of cardiac dysautonomia

Cardiac dysautonomia assessment was based on the four standardized tests conducted on a non-dialysis day. a) Beat-to-beat heart rate variation was assessed while subjects lying supine; they were instructed to breath six times per minute, and it was measured using an MT-50 Quartz Metronome (WITTNER GmbH & Co., Isny, Germany). Electrocardiogram strips were printed for one minute and labeled for inspiration (I) and expiration (E). Corrected QT interval (QTc) and expiration/inspiration R-R ratio (distance between two R waves) were noted. A difference in heart rate of fewer than 10 beats per minute and expiration/inspiration R-R ratio of <1.17 was considered abnormal. b) In the assessment of heart rate response to Valsalva maneuver, the subjects were directed to exhale forcibly into the mouthpiece of an in-house custom-made mercury manometer, exerting a pressure of 40 mm Hg for 15 seconds. A ratio of longest to the shortest R-R interval of <1.2 was scored abnormal. c) Heart rate response to standing was calculated by instructing the subjects to stand up and measuring the R-R interval at beats 15 and 30 after standing up. A 30:15 ratio of <1.03 was graded as an abnormal test. d) Systolic blood pressure response to standing was assessed by measuring the blood pressure manually with a sphygmomanometer to ascertain the difference between systolic blood pressure before and two minutes after standing. A fall of 20 mm Hg systolic blood pressure was considered abnormal. Finally, the criteria to diagnose CAN were based on abnormal results for at least two of the four tests.

SCD was defined as the death occurring from a cardiac cause in less than one hour of symptom onset in a participant, without any other pre-existing fatal condition, determined by history and medical records. In case of deaths outside the medical center, the mode of death was confirmed by contacting the surviving next-of-kin enlisted in the dialysis chart by the participant.

Ethics and consent

The research protocol was approved by the institutional review board of Allama Iqbal Medical College, Jinnah Hospital, Lahore, Pakistan. All patients gave written informed consent before participating in the study, which was conducted according to the principles of the Declaration of Helsinki as revised in Seoul in 2008.

Statistical analysis

Statistical analysis was performed using the SPSS 17.0 (Statistical Package for the Social Sciences) (IBM SPSS, Chicago, Illinois). Quantitative variables, such as age, were presented in the form of means ± standard deviations. Qualitative variables, such as sex, CAN etc. were demonstrated by frequency and percentages. Subject characteristics were compared with the use of the chi-square or Fisher's exact test for categorical variables while a non-parametric (Mann-Whitney U) test was used for continuous variables. A p-value <0.05 was considered statistically significant for all analyses at a 95% confidence interval (CI).

## Results

Twenty patients who were physically unfit for CAN testing were excluded. These patients were excluded on the basis of various causes. Out of 20, eleven (55%) patients were unable to stand, eight (40%) patients were unable to perform Valsalva maneuver, and one (5%) patient was unable to follow commands due to poor comprehension/mild cognitive limitation. A total of 50 participants completed the study protocol. Out of 50 patients, 26 (52%) were male with median age of 44 years (range, 15 to 72 years). Diabetes was present in 16 (32%) patients. At baseline, 44 (88%) patients were found to have cardiac dysautonomia. Resting tachycardia was present in seven (14%) patients. The median E:I ratio was 1.02 (range, 0.91 to 1.38). An abnormal heart rate response to deep breathing (E:I ratio ≤1.17) was observed in 47 (94%) patients with an E:I ratio below 1.0 seen in 10 (20%) patients. The median longest to shortest R-R ratio during Valsalva maneuver was 1.07 (range, 0.85 to 1.60). Abnormal Valsalva ratio (longest to shortest R-R ratio <1.2) was observed in 45 (90%) patients. The median 30th to 15th R-R ratio on standing was 1.0 (range, 0.79 to 1.25). An abnormal 30:15 ratio (i.e. < 1.03) was seen in 34 (68%) patients. Postural hypotension (postural drop in systolic blood pressure >20 mm Hg) was observed in five (10%) patients. Forty-four (78%) patients fulfilled our criteria for the diagnosis of CAN. We found four abnormal tests in nine (18%) patients, three abnormal tests in 22 (44%) patients, and two abnormal tests in 13 (26%) patients. Six (12%) patients did not have CAN at the baseline assessment (Table 1).

**Table 1:** Baseline characteristics of patients *E:I ratio (ratio of duration of inspiration to duration of expiration) †R-R ratio (Distance between two R waves of adjacent QRS complexes) NYHC, New York Heart Association Functional Classification

Characteristics	Number (%)
Total patients	50
Age (median) years	44
Males	26 (52%)
Females	24 (48%)
Diabetes mellitus	16 (32%)
Cardiac dysautonomia	44 (88%)
Resting tachycardia	7 (14%)
NYHC class I/II	33 (66%)
NYHC class III/IV	17 (34%)
Abnormal heart rate response to deep breathing (E:I ratio^*^ ≤1.17)	47 (94%)
Abnormal Valsalva ratio (longest to shortest R-R ratio^†^ <1.2)	45 (90%)
An abnormal 30:15 R-R ratio (i.e. < 1.03)	34 (68%)
Postural hypotension	5 (10%)

**Figure 1:**
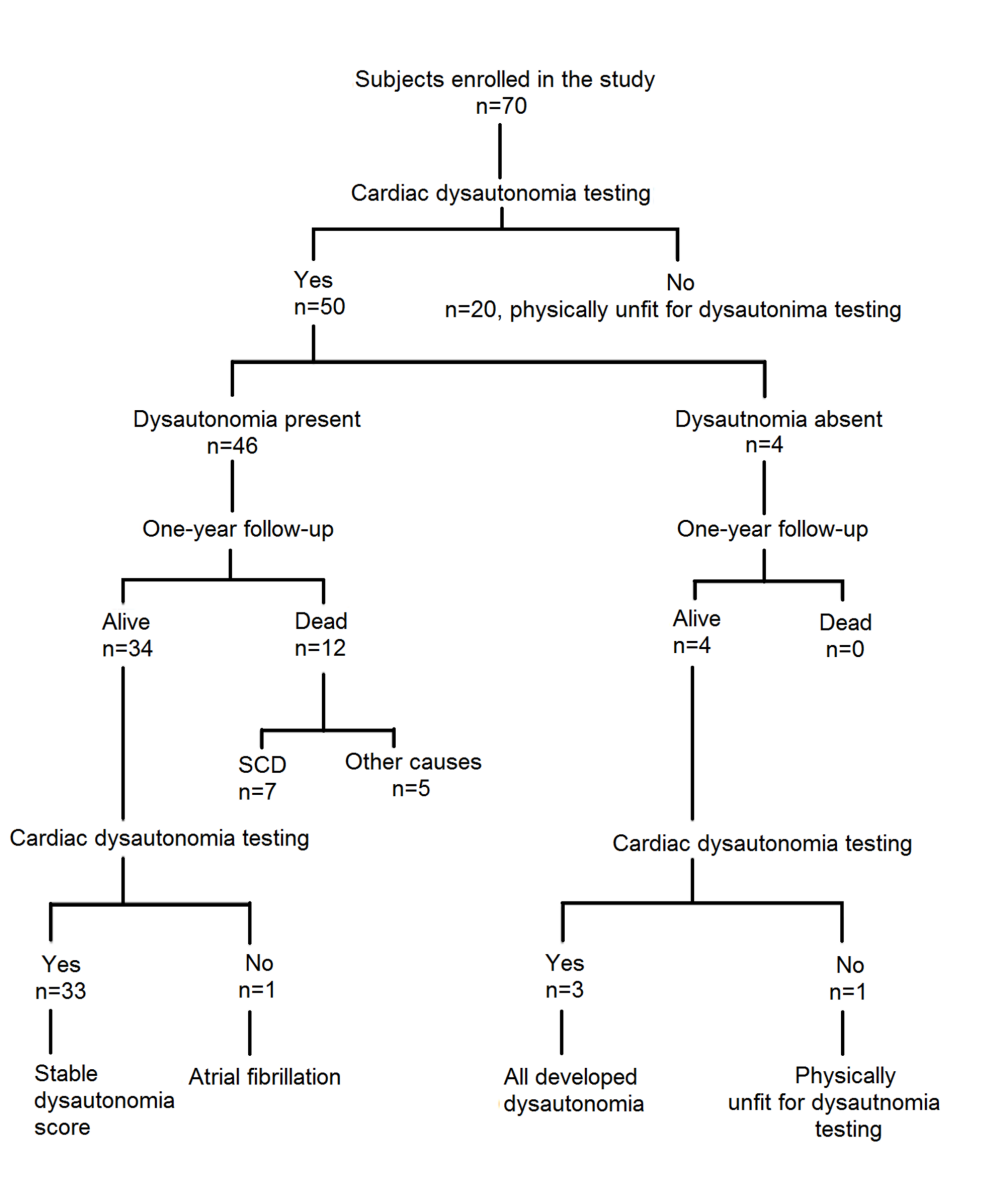
A flowchart diagram summarizing the data and results of patients included in our study SCD - sudden cardiac death

At the one-year follow-up, out of 50 participants included in our study, 38 (76%) patients were alive. Two patients of 38 were excluded from analysis. One patient developed atrial fibrillation and another was physically unable to perform Valsalva maneuver. Of 12 (24%) deceased patients, seven patients died due to SCD. Pneumonia, sepsis, and hypertensive encephalopathy were reported as causes of death in rest of the five patients. A significantly higher number of patients who died of any cause had abnormal results of all four tests for CAN as compared to patients who were alive at the end of one year (42% [5 of 12] patients vs. 10.5% [4 of 38], respectively; risk ratio 6.07, 95% CI 1.29-28.49; p-value, 0.02). Similarly, abnormality of all four test results was significantly higher in patients who had SCD as compared to those who were alive at the end of one year (43% [3 of 7] patients vs. 10.5% [4 of 38] respectively; risk ratio 6.37, 95% CI 1.03-39.36; p-value 0.04). Of the remaining six (12%) patients who did not have CAN at the baseline, five developed this condition at the end of one year. All seven patients who died of SCD had three abnormal CAN tests at the baseline. Rest of the patients with baseline cardiac dysautonomia had stable dysautonomia scores. Age, sex, diabetes status and duration of dialysis had no effect on dysautonomia score or mortality on regression analysis (Figure 1).

## Discussion

**Figure 2:**
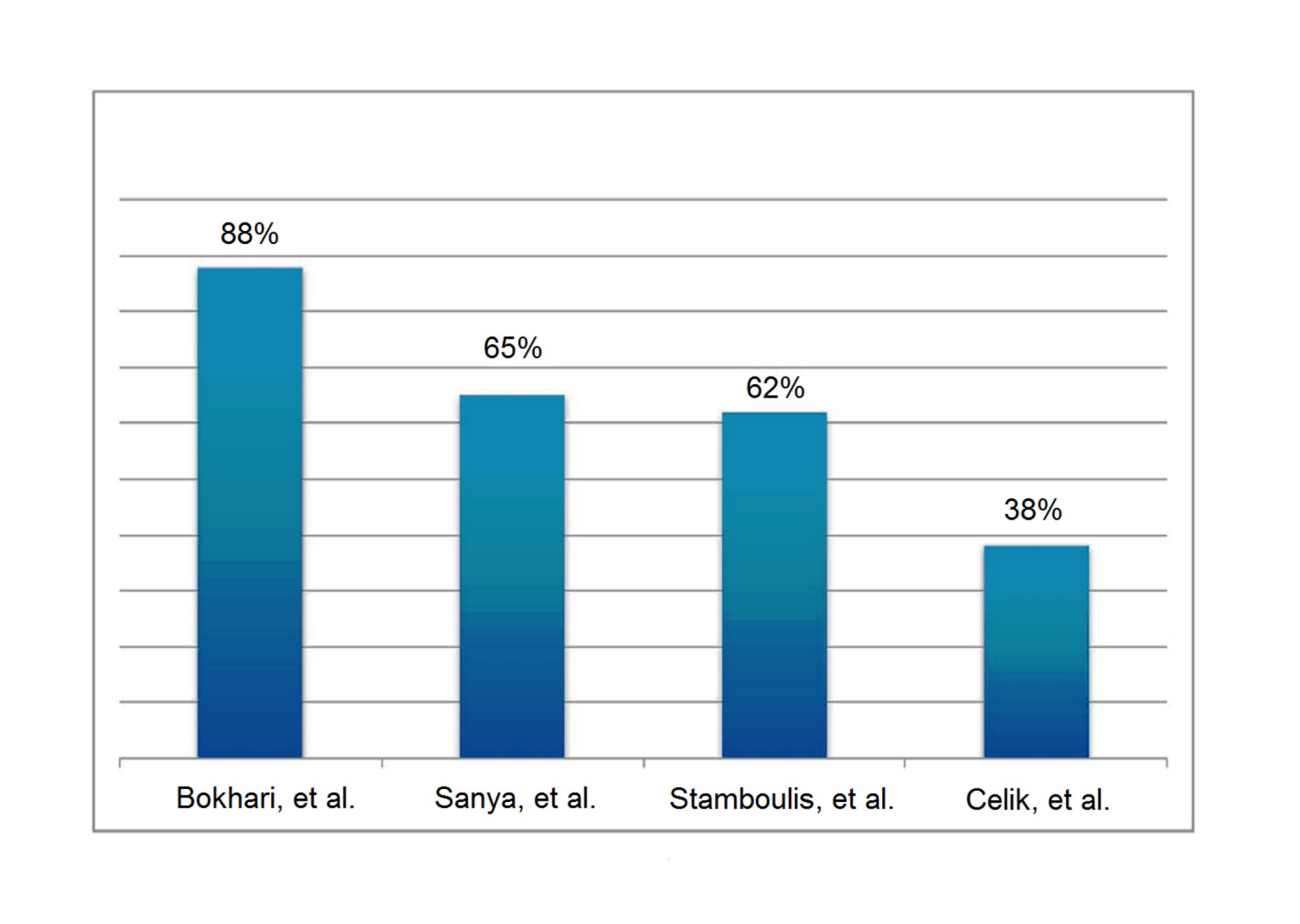
The cardiac dysautonomia incidence previously determined by three different research groups and its comparison with the prevalence observed in our study

The prevalence of CAN on the baseline testing in our study was remarkably high, which was independent of the diabetic status of the participants. Few studies found a varied frequency of CAN in the ESRD population ranging from 38% to 65%; however, the prevalence of CAN in our ESRD population was 88% [9-11] (Figure 2).

In our study, the reasons for the higher prevalence could be poor pre-ESRD care, late initiation of hemodialysis, and a significant number of associated comorbid conditions. This issue warrants further investigation to confirm the individual contribution of these probable causes to the relatively higher regional prevalence of CAN in patients with ESRD.

CAN has frequently been investigated in patients with diabetes. Silent myocardial ischemia and dysfunction, CV instability during or after surgery, stroke, the progression of renal disorders, and the appearance of foot ulcers in diabetes have been associated with CAN [12-14]. Therefore, it was considered as a probable pathogenetic mechanism behind increased mortality in patients with diabetes mellitus. CAN-related morbidity and mortality have also been researched in other diseases, including familial dysautonomia [15], Ehlers-Danlos syndrome [16], chronic obstructive pulmonary disease [17], liver cirrhosis [18], hypertension and hyperuricemia [19]. Furthermore, it was shown to predict all-cause mortality and sudden death in patients with chronic heart failure following ischemic or idiopathic dilated cardiomyopathy [20].

The present study designated CAN as an independent risk factor for all-cause and CV mortality in patients with ESRD. Several studies have implicated uremia as a major factor in the pathogenesis of cardiac dysautonomia in CKD, independent of diabetes [21]. Therefore, intensive dialysis and kidney transplantation improve CAN by correcting underlying uremic derangements [22]. In the patient population on dialysis, increased incidence of arrhythmias and SCD attributable to CAN has previously been documented. However, diabetes, hypertension, uremic dyslipidemia, and other conventional risk factors were unable to precisely differentiate the risk of SCD in patients with ESRD [23,24]. United States Renal Data System (USRDS) reports that 27% of all-cause mortality is related to SCD, which is in line with the findings of the HEMO and 4D trials [25].

The CAN testing in this study revealed that there was a significantly higher percentage of all four abnormal cardiac dysautonomia tests amongst patients who died of SCD, or death due to any cause at the end of one year. This shows that the high dysautonomia scores are associated with a higher risk of all-cause mortality as well as SCD. Furthermore, CAN has previously been associated with nocturnal hypoxemia, concentric hypertrophy, and remodeling considering it as the potential mechanism whereby hypoxemia triggers CV events in patients with ESRD [26]. At the same time, it is observed that peripheral sensorimotor neuropathy and degree of CAN have no association in patients on chronic hemodialysis [11]. Therefore, it indicates the need for discrete evaluation of CAN in patients with ESRD on dialysis, even if they have minimal or advanced peripheral neuropathy. It is also notable that in patients with ESRD who develop CAN, the parasympathetic nervous system involvement slightly supersedes the sympathetic nervous system and these patients frequently develop left ventricular hypertrophy due to reduced vagal tone [11].

In patients undergoing hemodialysis three times weekly, abnormal beat-to-beat variation was associated with increased mortality, particularly in patients with baseline beat-to-beat variability of 22 per minute. However, literature is scarce on the impact of the CAN in predicting long-term all-cause and CV mortality in ESRD patients. Few studies have implicated abnormal heart rate variability parameters as predictive of CV or all-cause mortality in dialysis patients [27-29]. Although heart rate variability has emerged as a simple method to evaluate autonomic nervous system function and has been used in several clinical trials, it still needs standardization. Furthermore, it is worth mentioning that the diagnosis of CAN requires at least two abnormal test results of the four tests performed for cardiac dysautonomia assessment.

Recently, Doulgerakis et al. prospectively evaluated CAN for CV and all-cause mortality in a cohort of 123 patients. At the five-year follow-up, the presence of CAN was found to be an independent predictor of all-cause and CV mortality. In addition to CAN, this study revealed that age, serum triglycerides, and left ventricular ejection fraction were independent risk factors for all-cause mortality in ESRD patients [30]. The results of our study add clinical evidence establishing cardiac dysautonomia testing as an effective risk stratification modality in patients with ESRD. Furthermore, our findings underscore the importance of CAN as a CV mortality contributor in patients with ESRD on hemodialysis. This prompts future research to investigate the pathogenesis of CAN in these patients that can help to curate potential treatment options for this abnormality.

This study has several limitations. The number of participants was relatively small. A large multicenter study will help in better understanding the frequency of CAN and incidence of SCD in patients on maintenance hemodialysis. We did not measure serum cholesterol, triglycerides or cardiac-function monitoring parameters in patients with ESRD recruited in our study. Therefore, we could not determine how much did these parameters contribute to the risk of SCD.

## Conclusions

The prevalence of CAN was significantly higher in patients with ESRD undergoing hemodialysis in our population. This study implicates CAN as an independent risk factor for all-cause and CV mortality in patients with ESRD. It emphasizes that CAN should be assessed and used for the risk stratification for SCD in such patients. 
